# Effect of Bone Metastasis Cancer Board on Spinal Surgery Outcomes: A Retrospective Study

**DOI:** 10.3390/medicina59122087

**Published:** 2023-11-28

**Authors:** Kunihiko Miyazaki, Yutaro Kanda, Yoshitada Sakai, Ryo Yoshikawa, Takashi Yurube, Yoshiki Takeoka, Hitomi Hara, Toshihiro Akisue, Ryosuke Kuroda, Kenichiro Kakutani

**Affiliations:** 1 Department of Orthopedic Surgery, Kobe University Graduate School of Medicine, Kobe 650-0017, Japan; 2 Division of Rehabilitation Medicine, Kobe University Graduate School of Medicine, Kobe 650-0017, Japan; 3 Department of Rehabilitation Science, Kobe University Graduate School of Health Sciences, Kobe 654-0142, Japan

**Keywords:** bone metastasis, spinal metastasis, cancer board, bone management, multidisciplinary treatment, multidisciplinary cancer conference, spinal neoplasms, orthopedics

## Abstract

*Background and Objectives*: Bone metastasis cancer boards (BMCBs) focusing on the management of bone metastases have been gathering much attention. However, the association of BMCBs with spinal surgery in patients with spinal metastases remains unclear. In this retrospective single-center observational study, we aimed to clarify the effect of a BMCB on spinal metastasis treatment. *Materials and Methods*: We reviewed consecutive cases of posterior decompression and/or instrumentation surgery for metastatic spinal tumors from 2008 to 2019. The BMCB involved a team of specialists in orthopedics, rehabilitation medicine, radiation oncology, radiology, palliative supportive care, oncology, and hematology. We compared demographics, eastern cooperative oncology group performance status (ECOGPS), Barthel index (BI), number of overall versus emergency surgeries, and primary tumors between patients before (2008–2012) and after (2013–2019) BMCB establishment. *Results:* A total of 226 patients including 33 patients before BMCB started were enrolled; lung cancer was the most common primary tumor. After BMCB establishment, the mean patient age was 5 years older (*p* = 0.028), the mean operating time was 34 min shorter (*p* = 0.025), the mean hospital stay was 34.5 days shorter (*p* < 0.001), and the mean BI before surgery was 12 points higher (*p* = 0.049) than before. Moreover, the mean number of surgeries per year increased more than fourfold to 27.6 per year (*p* < 0.01) and emergency surgery rates decreased from 48.5% to 29.0% (*p* = 0.041). Patients with an unknown primary tumor before surgery decreased from 24.2% to 9.3% (*p* = 0.033). Postoperative deterioration rates from 1 to 6 months after surgery of ECOGPS and BI after BMCB started were lower than before (*p* = 0.045 and *p* = 0.027, respectively). *Conclusion:* The BMCB decreased the emergency surgery and unknown primary tumor rate despite an increase in the overall number of spinal surgeries. The BMCB also contributed to shorter operation times, shorter hospital stays, and lower postoperative deterioration rates of ECOGPS and BI.

## 1. Introduction

With recent progress in cancer treatment, the number of patients with bone metastases has rapidly increased [1]. Since patients with bone metastases require treatment for both the metastases and the primary tumor, it is often difficult for the primary department to treat these cases alone [2,3,4]. Generally, bone metastasis is treated by orthopedic surgery, radiation therapy, and chemotherapy [2,3,5]. As most patients receive a combination of these therapies, multidisciplinary treatment and cooperation are essential [2,3,6,7]. Thus, bone metastasis cancer boards (BMCBs) that focus on managing bone metastasis and involve a team of specialists in oncology, palliative care, radiotherapy, orthopedics, radiology, and physiatry to provide multidisciplinary treatment have recently become widespread.

The spine is the most common site of bone metastases. Spinal metastases mostly progress asymptomatically in the early stages. Moreover, approximately 10–20% of patients with spinal metastases have significantly reduced performance status (PS) and activities of daily living (ADL) due to serious symptoms, such as neurological dysfunction and intractable pain, making it sometimes challenging to continue therapies for the primary tumor. Therefore, treating spinal metastases is crucial for patients with this condition [2,3,4,8,9,10].

Although some clinical studies have reported the importance of councils, conferences, and multidisciplinary teams, few have focused on spinal metastases surgery [3,7]. A randomized controlled study demonstrated that surgery for spinal metastases following postoperative radiotherapy was superior to radiotherapy alone in terms of ambulatory status, regaining walking ability, ambulatory duration, and survival [5]. Spinal metastasis surgery can also improve patients’ PS and quality of life for at least 6 months postoperatively. However, the association between BMCBs and surgical outcomes in patients with spinal metastases remains unclear. Therefore, we designed this study to elucidate the impact of BMCB on spinal surgery.

## 2. Materials and Methods

### 2.1. Ethics Statement

This study was approved by the Institutional Review Board of Kobe University Hospital, Japan. Written informed consent was obtained from each patient according to the principles of the Declaration of Helsinki and the Japanese laws and regulations.

### 2.2. Characterization of the BMCB

Our BMCB was established in 2013, primarily by spine surgeons and physiatrists. Our concept for the bone management was well accepted by the hospital administration, medical staff, and other specialists and the BMCB gained recognition within our institution. Our BMCB mainly consisted of doctors from orthopedics (spine surgeons and oncologists), rehabilitation medicine, radiation oncology, radiology (interventional radiology specialists), palliative supportive care, oncology, and hematology, as well as those from the main medical departments. Additionally, our BMCB included nurses, pharmacists, and physical therapists involved in palliative care. All hospital staff could participate in our BMCB conference without any training and there was no voting system for decision making. All members were free to express their opinions, which were then organized to guide the optimal treatment. Meetings were held every 3 weeks and lasted approximately 60 min depending on the number of cases.

BMCB members, primarily spine surgeons and oncologists, enrolled patients with metastatic spinal tumors in the database during daily outpatient visits or rounds. The patients were followed up in the orthopedics, radiation oncology, and rehabilitation departments. Patient data, including imaging findings, treatment history, PS, and ADL, were continuously recorded in the BMCB database, which was then used to confirm the diagnosis, consider surgical treatment, adapt radiation therapy, assess the necessity for rehabilitation, determine the settings for orthotic treatment, and assess the level of rest.

### 2.3. Clinical Study Design

We retrospectively reviewed consecutive patients who underwent palliative posterior decompression and/or instrumentation surgery for metastatic spinal tumors at our hospital between 2008 (an electronic medical record system was introduced) and 2019. Metastases were diagnosed using radiography, computed tomography, magnetic resonance imaging (MRI), bone scintigraphy, positron emission tomography, and histological evaluation of needle biopsies.

Surgical indications were progressive neuropathy and intractable pain refractory to conservative therapy, including opioid use. Emergency surgery was defined as surgery occurring within 48 h of diagnosis in patients with advanced neuropathy. The contraindications to surgery include impaired consciousness due to brain metastasis, dementia, and inability to make decisions. Consequently, 226 patients with spinal cord metastases were eligible for surgery. All surgeries were performed using a posterior approach. The surgeon made a comprehensive decision regarding the choice of surgical procedure based on the Bilsky Cord Compression Score [11] and the Spinal Instability Neoplastic Score [12] (SINS). Generally, patients with tumor-induced spinal canal stenosis (Bilsky Cord Compression Score ≥1) underwent palliative decompression, those with instability (SINS of ≥7) underwent posterior fixation with instrumentation, and those with both conditions underwent laminectomy and instrumentation. We compared demographic characteristics, the severity of spinal metastasis (Katagiri [13] and Tokuhashi [14] scores, Frankel classifications [15]), PS (eastern cooperative oncology group performance status [ECOGPS]) grade), ADL (Barthel index [BI]), postoperative deterioration rate of ECOGPS and BI, surgical data (surgeries performed, operation time, blood loss, and length of hospital stay), and primary cancer site before and after BMCB establishment.

Clinical follow-up was conducted at 1, 3, and 6 months postoperatively and every 3 months thereafter. Improvement or deterioration in each subjective health status was defined as a change in at least one level on the ECOGPS and at least 10 points on the BI. Changes from the baseline to 1 month and from 1 month to 6 months postoperatively were investigated to clarify the early- and mid-term effects of multidisciplinary treatment, respectively. Midterm assessments are more strongly affected by multidisciplinary treatment after surgery whereas early assessment more directly reflects the impact of the surgery itself. Given these characteristics, during the midterm evaluation, we compared the number of patients with deteriorated ECOGPS or BI scores with those who improved or maintained their ECOGPS or BI scores. All surviving patients who were unable to attend the outpatient clinic were telephoned to obtain the latest follow-up information. For patients who died, information was obtained from their families or the hospital to which they were transferred.

### 2.4. Statistical Analysis

All statistical analyses were performed using SPSS (version 13.0; SPSS, Inc., Chicago, IL, USA), with significance set at *p* < 0.05. Mann–Whitney U tests (continuous variables) and the Chi-square or Fisher’s exact test (categorical variables) were used to compare preoperative and surgery-related factors between the two groups before (2008–2012) and after (2013–2019) the establishment of BMCB. Changes over time between the two groups were identified using the Kruskal–Wallis test.

## 3. Results

### 3.1. Clinical Characteristics of Patients Pre- versus Post- BMCB Implementation

The following surgeries were performed on the included patients via a posterior approach: posterior decompression alone (*n* = 20), posterior instrumentation alone (*n* = 46), and posterior decompression and instrumentation (*n* = 160). The demographic data comparing 2008–2012 and 2013–2019 (pre- and post-BMCB establishment) are presented in Table 1. In post-BMCB establishment, the mean age was 5 years higher (*p* = 0.028) than pre-BMCB. The mean operating time and hospital stay post-BMCB establishment were significantly shorter, by 28 min and 29.5 days, respectively, than those in the pre-BMCB group (*p* = 0.025 and *p* < 0.001, respectively). The mean BI was 56.0 at post-BMCB whereas it was 44.1 at pre-BMCB (*p* = 0.049). Lung cancer was the most common primary tumor, followed by kidney and breast cancers (Table 2). A higher proportion of patients with unknown primary tumors at the time of surgery was observed in the pre-BMCB group (24.2%) than in the post-BMCB group (9.3%) (*p* = 0.033).

### 3.2. Transition of the Number of Total Spinal Surgeries and Emergency Surgeries

**Figure 1:**
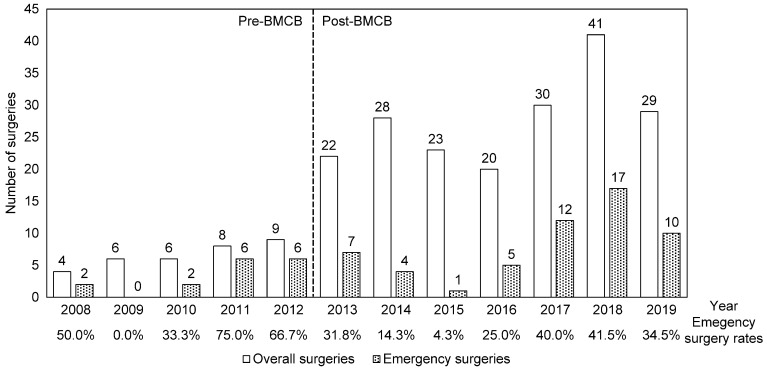
Number of overall and emergency surgeries per year. The period of 2008–2012 was before the establishment of the bone metastasis cancer board, while that of 2013–2019 was after. Total surgeries were compared using the Mann–Whitney U test (*p* = 0.003), while emergency surgeries were compared using Fisher’s exact test (*p* = 0.416).

The total number of surgeries and percentage of emergency surgeries performed in the two groups are shown in Figure 1. The mean number of spine surgeries per year increased from 6.6 pre-BMCB establishment to 27.6 post-BMCB establishment (*p* < 0.001), while the percentage of emergency surgeries decreased from 48.5% pre-BMCB establishment to 29.0% post-BMCB establishment (*p* = 0.041).

### 3.3. PS and ADL

There was no difference in the preoperative PS median between the pre- and post-BMCB establishment. In both groups, the PS median improved from three at the baseline to two at 1 month and one at 3 months postoperatively (Table 3). BMCB establishment was associated with an increased baseline BI (*p* = 0.049) (Table 3). Throughout the postoperative follow-up time points within 6 months, the mean of BI post-BMCB establishment was approximately 10 points higher than pre-BMCB establishment, although the difference was not significant.

The postoperative individual chronological changes in ECOGPS and BI are shown in Table 4. Approximately 70% of patients showed improvement at 1 month postoperatively compared to baseline, as evaluated by ECOGPS and BI, regardless of BMCB establishment. The rates of deterioration in ECOGPS and BI from 1 month to 6 months postoperatively were significantly lower post-BMCB establishment than pre-BMCB establishment (*p* = 0.045 and *p* = 0.027, respectively) whereas those from the baseline to 1 month postoperatively showed no significant difference between the two groups.

### 3.4. Neurological Function

There was no significant change in the ratios of Frankel grades D and E to A–C post-BMCB establishment (pre-BMCB D and E, 57.6%; post-BMCB D and E, 58.5%) (Table 1). Almost all patients with Frankel grade E at baseline, with (96.0%) or without (100.0%) BMCB, improved or at least maintained their neurological function at the final follow-up (Table 5). Meanwhile, the postoperative deterioration rate of Franke grade (more than one grade) was rarely observed (9/226, 4.0%). There was no significant difference between the pre-BMCB (0%, 0/33) and post-BMCB establishment (4.7%, 9/193) (*p* = 0.363).

## 4. Discussion

We investigated the effect of BMCB on surgical outcomes for spinal metastases, which to our knowledge, is the first study of this kind. This study elucidated that BMCB has some beneficial effects on spinal surgery.

In this study, there were no significant differences in preoperative clinical characteristics between the pre- and post-BMCB establishment groups, except for age and unknown primary tumor rate. As this study involved a consecutive case series, our aged society might have contributed to an increased proportion of the older population post-BMCB establishment. We also surmise that with the rapid advancements in medical technology and the range of treatments for geriatric patients, the proportion of elderly patients has also increased. Regarding the primary tumor site, lung cancer was the most frequent primary source of metastatic spinal tumors in our study, which is consistent with a previous report on other Asian populations by Wright et al. [1]. In addition, the number of cases with unknown primary sites of spinal metastasis at the time of surgery decreased post-BMCB establishment, possibly due to improvements in diagnostic techniques, increased awareness of spinal metastases in other departments, improved communication between departments, and an increased number of patients diagnosed and referred to spine surgeons before their neurological symptoms appeared or worsened. In recent publications, the incidence of tumors of unknown primary cause has shown a decreasing trend. The decline can be attributed to the decrease in the incidence of lung cancer: the most commonly reported unknown primaries, higher rate of identification based on advanced diagnostic methods, and alteration of sociodemographic factors such as a smoking habit that might be associated with the cases of tumors with unknown primary cause [16,17]. As the trends of an increasing number of elderly patients and a decreasing rate of unknown primary tumor rates are expected to continue, the present study is likely to add valuable insights into evolving scenarios of clinical oncology.

With the establishment of the BMCB in 2013, the number of spinal surgeries increased in our hospital. This may be due to the increased number of patients, increased sensitivity of diagnostic tests, and improved treatment of patients with advanced cancer stages [1,3]. Additionally, the BMCB may have directly contributed as there were more opportunities for communication between professionals. In fact, before the BMCB was established, some patients with spinal metastases might have been referred to spinal surgeons after developing serious symptoms and missing the optimal timing for surgical intervention. Thus, BMCB might serve as a safety net to prevent missed surgical opportunities.

Additionally, emergency surgery rates decreased after the BMCB establishment. Since BMCB provides an opportunity to share patient information and discuss therapeutic strategies before patients with spinal metastases develop serious symptoms, therapeutic interventions, such as prophylactic surgery and radiotherapy, might contribute to the decrease in emergency surgery rates. In fact, the preoperative radiotherapy rate increased after BMCB initiation, although the difference was not statistically significant. Failure to diagnose and treat are factors associated with litigation risk, which is particularly high during emergency surgery [18]. In emergency surgery, diagnostic errors and poor communication among doctors, patients, and medical personnel can lead to postoperative complications. Johnson and Knobf [19] reviewed the advantages of surgery for impending pathological fractures over actual fractures, including improved patient outcomes, fewer complicated surgical procedures, and shorter postoperative hospital stays. Similarly, Ristevski et al. [20] reported better overall survival in patients who underwent prophylactic stabilization than in those who underwent pathological fracture fixation for femoral metastatic lesions. We believe that an aging population and the increase in the number of cancer patients have led to an increase in the absolute number of patients with metastatic tumors which in turn has resulted in an increase in the number of emergency surgeries, a problem that cannot be overlooked. Our hospital receives a considerable number of patients in need of emergency surgeries including those with severe paralysis who are referred by other hospitals and many of these patients are past the best time for surgical intervention. Consequently, BMCB, which reduces the relative rate of emergency surgeries, may reduce the burden on spine surgeons.

We observed shorter operating times after the BMCB was established, which can be partly due to the learning curve. However, we cannot ignore the possibility of improvement in preoperative preparation owing to a decrease in emergency surgery by BMCB, e.g., preoperative endovascular embolization can minimize perioperative bleeding from tumor tissue in cases where the primary tumor was preoperatively detected as hypervascular. Additionally, the minimally invasive percutaneous pedicle screw system may have contributed to these results. Posterior instrumentation without decompression has increased after the BMCB establishment.

We also observed shorter hospitalization after the BMCB establishment. Although medical staff tend to underestimate the effects of hospitalization, it is an important aspect in terms of patients’ quality of life and medical costs. Uei et al. [7] showed that a multidisciplinary approach to conventional posterior decompression and fixation treatment combined with postoperative treatments such as chemotherapy, radiotherapy, and bone-modifying agents facilitated early home discharge. The reason for the shorter hospital stays may be due to changes in Japan’s reimbursement system but it may also be due to smoother cooperation among medical departments and quicker rehabilitation after treatment. Regardless of BMCB, spinal surgery immediately improved patients’ ECOGPS and BI scores. We also analyzed these results from an individual standpoint and found that the BMCB establishment did not affect early outcomes, mainly due to the spinal surgery itself (changes from baseline to 1 month postoperatively). However, in the mid-term, the number of patients who experienced deterioration in ECOGPS and BI decreased significantly. This may have been due to a higher preoperative BI after BMCB establishment, which potentially resulted in better postoperative courses. However, these results also suggest that multidisciplinary approaches using BMCB, including rehabilitation or optimized postoperative therapies, might help patients with spinal metastases further improve or maintain their PS and ADL from a mid-term perspective.

Multidisciplinary team meetings for cancer treatment (known as cancer or tumor boards) reportedly led to significant changes in the way cancer patients are assessed and managed [21]. In a systematic review, Coory et al. [22] reported a reduced median time from presentation to first treatment, time from presentation to surgery, and average number of days from cancer diagnosis to treatment. Dickhoff and Dahel [23] also suggested that the establishment of multidisciplinary cancer team meetings should be considered equivalent to other medical interventions, such as the introduction of novel medications, as multidisciplinary cancer teams enable improved diagnosis (including staging) and treatment (including better adherence to guidelines and higher recruitment rates for clinical trials) and, consequently, longer overall patient survival. However, the effect of meetings on the assessment and management of patients is influenced by the cancer type and stage, structural and functional factors, and expertise of the participating professionals [21]. Therefore, it is an important finding that BMCB, which is specific to bone metastases and not each cancer type, provided various benefits in this study.

This study has several limitations. First, a relatively small number of pre-BMCB establishment cases were used as controls. We acknowledge that the lack of a more extensive control group limits the robustness of the comparison. Second, because this was a retrospective study, the presence of bias and confounding factors were considered limiting factors, although most preoperative demographic and clinical characteristics did not differ between the two groups. Recent progress in cancer treatment, including stereotactic radiosurgery, molecularly targeted drugs, and especially immune checkpoint inhibitors, may affect the outcomes in patients with cancer as a confounding factor. Notably, immune checkpoint inhibitors have emerged as a promising therapy against lung cancer [24]. With the gradual progression of the therapeutic options for managing and treating cancer, prognosis and treatment strategies often evolve, highlighting the need to update the role of BMCB. BMCB, which involves opinions from a multidisciplinary perspective, has the advantage of being able to flexibly respond to such changes. However, the effectiveness of BMCB may vary depending on the availability of resources, the patient population, and the physicians involved. Therefore, the generalizability of this study may be limited. However, the BMCB might play a more important role today as treatment decision making becomes more diverse and complex.

## 5. Conclusions

In conclusion, BMCB focuses on the management of bone metastases and decreases the incidence of emergency spinal surgery despite an increase in the overall number of spinal surgeries. BMCB also helped clinicians to detect the primary tumor type and contributed to shorter hospital stays and lower postoperative deterioration rates in ECOGPS and BI.

## Figures and Tables

**Table 1:** Baseline demographic and clinical characteristics of patients.

Variable	Total (*n* = 226)	Pre-BMCB (*n* = 33)	Post-BMCB (*n* = 193)	*p*-Value
Mean age, years (range)	66.0 (24–92)	61.8 (31–84)	66.8 (24–92)	0.028 ‡
Male sex, n (%)	140 (61.9%)	22 (66.7%)	118 (61.1%)	0.690 *
Mean Katagiri score (range)	5.0 (0–9)	5.2 (1–8)	5.1 (0–9)	0.786 ‡
Mean Tokuhashi score (range)	6.1 (1–13)	6.1 (2–12)	6.1 (1–13)	0.746 ‡
Mean SINS (range)	10.5 (2–18)	9.7 (3–16)	10.7 (2–18)	0.069 ‡
Pre-radiation, n (%)	61 (27.0%)	5 (15.2%)	56 (29.0%)	0.136 *
Pre-chemotherapy, n (%)	104 (46.0%)	16 (48.5%)	88 (45.6%)	0.851 *
Mean operating time, min (range)	201 (59–1735)	229 (53–440)	196 (59–1735)	0.025 ‡
Mean blood loss, mL (range)	327 (0–2500)	295 (0–1400)	332 (0–2500)	0.748 ‡
Mean hospital stay, days (range)	25.3 (0–430)	54.8 (10–353)	20.3 (0–430)	<0.001 ‡
Operation				
Posterior stabilization	46	1	45	
Posterior decompression	20	9	11	
Posterior decompression and stabilization	160	23	137	
ECOGPS grade, n				0.127 †
PS1	20	2	18	
PS2	35	3	32	
PS3	74	9	65	
PS4	97	19	78	
Mean BI (range)	54.2 (0–100)	44.1 (5–100)	56.0 (0–100)	0.049 ‡
Frankel classification, n (%)				>0.999 *
Grade A, B, and C	94 (41.6)	14 (42.4)	80 (41.5)	
Grade D and E	132 (58.4)	19 (57.6)	113 (58.5)	

* Fisher’s exact test; † Chi-square test; ‡ Mann–Whitney U test. Abbreviations: BMCB, bone metastasis cancer board; SINS, spinal instability neoplastic score; ECOGPS, eastern cooperative oncology group performance status; BI, Barthel index.

**Table 2:** Origin of metastases.

Organ	Number of Patients		
	Total	Pre-BMCB	Post-BMCB
Lung	37	2	35
Kidney	22	2	20
Breast	20	1	19
Liver	17	1	16
Thyroid	13	3	10
Malignant lymphoma	12	4	8
Colon	12	2	10
Multiple myeloma	11	0	11
Sarcoma	10	3	7
Prostate	9	1	8
Esophagus	4	0	4
Bladder	4	0	4
Malignant melanoma	4	0	4
Ovary	2	0	2
Uterus	2	0	2
Pancreas	2	0	2
Other	19	6	13
Unknown	26	8	18
Total	226	33	193
Percent of unknown (%)	11.5	24.2	9.3

Fisher’s exact test was used to identify the differences in unknown tumor rates between pre- and post-BMCB, *p* = 0.033. Abbreviations: BMCB, bone metastasis cancer board.

**Table 3:** ECOGPS and BI.

Variable	Pre-BMCB	Post-BMCB	*p*-Value
Median ECOGPS (IQR), (number of PS 0, 1, 2, 3, 4)			
Baseline	3 (3–4), (0, 3, 3, 9, 18)	3 (3–4), (0, 17, 32, 65, 79)	0.180
1 months	2 (1–3), (0, 11, 11, 10, 1)	2 (1–3), (4, 69, 54, 41, 21)	0.857
3 months	1 (1–2), (0, 16, 9, 4, 2)	1 (1–2), (5, 65, 28, 22, 5)	0.630
6 months	1 (1–2), (0, 16, 3, 3, 2)	1 (1–2), (5, 56, 15, 6, 1)	0.210
Mean BI (range)			
Baseline	44.1 (5–100)	56.0 (0–100)	0.049
1 month	68.5 (10–100)	76.0 (0–100)	0.075
3 months	72.1 (10–100)	84.3 (0–100)	0.134
6 months	81.9 (5–100)	92.9 (10–100)	0.086

The Kruskal–Wallis test was used. Abbreviations: BMCB, bone metastasis cancer board; ECOGPS, eastern cooperative oncology group performance status; IQR, interquartile range; BI, Barthel index; PS, performance status.

**Table 4:** Individual chronological changes in ECOGPS and BI scores.

**Early Effect (Changes from Baseline to Month Postoperatively)**	**Pre-BMCB**	**Post-BMCB**	***p-*Value**
ECOGPS, n (%)			
Improvement	25 (75.8%)	133 (70.4%)	
Unchanged	8 (24.2%)	51 (27.0%)	>0.999
Deterioration	0 (0%)	5 (2.7%)	
BI, n (%)			
Improvement	23 (69.7%)	130 (68.8%)	
Unchanged	10 (30.3%)	54 (28.6%)	>0.999
Deterioration	0 (0%)	5 (2.7%)	
**Mid-Term Effect (Changes from 1–6 Months Postoperatively)**	**Pre-BMCB**	**Post-BMCB**	***p-*Value**
ECOGPS, n (%)			
Improvement	9 (37.5%)	19 (23.2%)	0.045
Unchanged	10 (41.7%)	58 (70.7%)	
Deterioration	5 (20.8%)	5 (6.1%)	
BI, n (%)			
Improvement	9 (37.5%)	15 (18.3%)	0.027
Unchanged	10 (41.7%)	63 (76.8%)	
Deterioration	5 (20.8%)	4 (4.9%)	

A Chi-square test was used to identify differences in the rate of deterioration in ECOGPS between 1 and 6 months after surgery. Improvement or deterioration of subjective health status values was defined as a change in more than one level in the ECOGPS and more than 10 points in the Barthel index. Abbreviations: ECOGPS, eastern cooperative oncology group performance status; BI, Barthel index; BMCB, bone metastasis cancer board.

**Table 5:** Frankel grade variation before surgery to the last postoperative follow-up.

Pre-BMCB (*n* = 33)						Post-BMCB (*n* = 193)					
A–A	A–B	A–C	A–D	A–E	Total	A–A	A–B	A–C	A–D	A–E	Total
0	0	0	0	0	0	2	0	0	0	0	2
0.0%	0.0%	0.0%	0.0%	0.0%		1.0%	0.0%	0.0%	0.0%	0.0%	
B–A	B–B	B–C	B–D	B–E	Total	B–A	B–B	B–C	B–D	B–E	Total
0	0	1	2	1	4	0	3	4	0	0	7
0.0%	0.0%	3.0%	6.1%	3.0%		0.0%	1.6%	2.1%	0.0%	0.0%	
C–A	C–B	C–C	C–D	C–E	Total	C–A	C–B	C–C	C–D	C–E	Total
0	0	3	3	4	10	0	0	20	36	15	71
0.0%	0.0%	9.1%	9.1%	12.1%		0.0%	0.0%	10.4%	18.7%	7.8%	
D–A	D–B	D–C	D–D	D–E	Total	D–A	D–B	D–C	D–D	D–E	Total
0	0	0	3	8	11	0	1	6	17	35	59
0.0%	0.0%	0.0%	9.1%	24.2%		0.0%	0.5%	3.1%	8.8%	18.1%	
E–A	E–B	E–C	E–D	E–E	Total	E–A	E–B	E–C	E–D	E–E	Total
0	0	0	0	8	8	0	0	0	2	52	54
0.0%	0.0%	0.0%	0.0%	24.2%		0.0%	0.0%	0.0%	1.0%	26.9%	

The first letter corresponds to preoperative grade. The second letter of each pair corresponds to the grade obtained during the last postoperative follow-up.

## Data Availability

The data presented in this study are available on request from the corresponding author. The data are not publicly available due to privacy and ethical concerns.
